# Multifork chromosome replication in slow-growing bacteria

**DOI:** 10.1038/srep43836

**Published:** 2017-03-06

**Authors:** Damian Trojanowski, Joanna Hołówka, Katarzyna Ginda, Dagmara Jakimowicz, Jolanta Zakrzewska-Czerwińska

**Affiliations:** 1 Department of Molecular Microbiology, Faculty of Biotechnology, University of Wrocław, Wrocław, Poland; 2 Hirszfeld Institute of Immunology and Experimental Therapy, Polish Academy of Sciences, Wrocław, Poland; 3 Department of Biochemistry, University of Oxford, South Parks Road, Oxford OX1 3QU, UK

## Abstract

The growth rates of bacteria must be coordinated with major cell cycle events, including chromosome replication. When the doubling time (Td) is shorter than the duration of chromosome replication (C period), a new round of replication begins before the previous round terminates. Thus, newborn cells inherit partially duplicated chromosomes. This phenomenon, which is termed multifork replication, occurs among fast-growing bacteria such as *Escherichia coli* and *Bacillus subtilis*. In contrast, it was historically believed that slow-growing bacteria (including mycobacteria) do not reinitiate chromosome replication until the previous round has been completed. Here, we use single-cell time-lapse analyses to reveal that mycobacterial cell populations exhibit heterogeneity in their DNA replication dynamics. In addition to cells with non-overlapping replication rounds, we observed cells in which the next replication round was initiated before completion of the previous replication round. We speculate that this heterogeneity may reflect a relaxation of cell cycle checkpoints, possibly increasing the ability of slow-growing mycobacteria to adapt to environmental conditions.

Bacteria must precisely coordinate growth rate with major cell cycle events, including chromosome replication. The bacterial cell cycle can be divided into three periods: the time between cell division and the initiation of replication (**B period**); chromosome replication (**C period**); and the time between the termination of replication and the completion of subsequent cell division (**D period**)^1^. When the doubling time (Td) is shorter than the duration of chromosome replication (C period), a new round of replication begins before the previous round terminates. Thus, newborn cells inherit partially duplicated chromosomes. This phenomenon, termed multifork replication, is well described in fast-growing model organisms^1^,^2^,^3^,^4^,^5^,^6^,^7^,^8^,^9^,^10^,^11^ but has not previously been observed in slow-growing bacteria such as mycobacteria. Mycobacteria exhibit an unusual mode of cell elongation and division^12^,^13^,^14^,^15^. In contrast to *Escherichia coli* and *Bacillus subtilis*, they grow by polar extension and often divide asymmetrically to generate daughter cells that differ in size. Recent studies used DnaN-FP (FP stands for fluorescent proteins such as EGFP or mCherry) to mark the sliding clamp and enable visualization of DNA replication forks (replisomes) in mycobacteria^16^,^17^. In these studies, the appearance and disappearance of DnaN-FP foci indicate assembly and disassembly, respectively, of the replisome complex and are considered to correspond to the initiation and termination of DNA replication, respectively. These studies found that DNA replication occurs near the midcell and that the replisomes are highly dynamic, frequently splitting over a short distance and then merging back together. These prior reports also revealed that shortly after the initiation of chromosome replication, two copies of a newly replicated chromosomal origin (*oriC*) are bound by the segregation protein (ParB) and move towards the cell poles. Moreover, it was found that after mycobacteria complete chromosome replication, a new round of replication is initiated prior to cytokinesis of the mother cell (monitored using Wag31-GFP) and is completed in the daughter cells^12^. However, the processes involved in the multifork replication of mycobacteria have not yet been described.

## Results and Discussion

In our earlier time-lapse fluorescence microscopic (TLFM) analyses of single-cell replisome dynamics in *Mycobacterium smegmatis*, we noted that a subfraction of cells had more than two DnaN-FP foci^17^. Moreover, distances between individual DnaN-FP foci were frequently longer than the corresponding distances in cells with two fluorescent spots. The presence of widely separated DnaN foci could reflect that a sliding clamp is associated longer with the template (especially on lagging-strand Okazaki fragments), that DnaN-mediated DNA repair and recombination is underway^18^,^19^,^20^,^21^ or that chromosome replication has been re-initiated.

**Figure 1:**
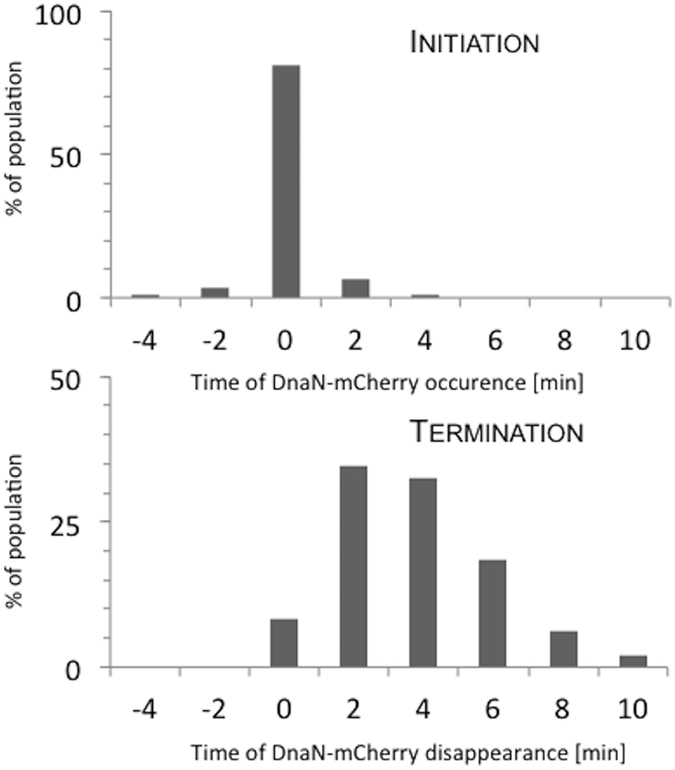
Kinetics of the appearance and disappearance of DnaN-mCherry foci with respect to alpha-EYFP foci. (top panel) Initiation of DNA replication; t = 0 indicates the simultaneous appearance of both replisome subunits. (bottom panel) Termination of DNA replication; t = 0 indicates the simultaneous disappearance of both replisome subunits (n = 53).

To assess whether the appearance of additional DnaN foci reflects replication reinitiation, we visualized the alpha (catalytic) subunit, which is directly involved in DNA synthesis. We constructed two strains: one encoding the DNA polymerase III alpha subunit fused with EYFP and one encoding the two replisome fluorescent markers, alpha-EYFP and DnaN-mCherry. In both strains, the fusion proteins were expressed from their native loci. A snap-shot analysis revealed that the localization pattern of alpha-EYFP was similar to those observed in the DnaN-FP strains (Supplementary Fig. 1a)^17^. To test whether fusion of the alpha subunit to EYFP disturbed DNA replication, we used TLFM to compare the cell cycle parameters of the analyzed fluorescent reporter strains. We found that the duration of DNA replication was shorter in the strain expressing the sliding clamp fused with mCherry (120 +/−9 min) than in those expressing the alpha subunit fused to EYFP or both fluorescent replisome markers (145 +/−10 min, Table 1). Overall, replication was slightly delayed in strains expressing the catalytic subunit fused to EYFP. However, we did not note any significant difference in the growth rates of the alpha and DnaN strains (see growth curves, Supplementary Fig. 1b). The lack of a difference in growth rate was most likely a consequence of the shortened B and/or D periods of the alpha-labeled strain (see Table 1). Next, we used TLFM to simultaneously track the dynamics of both replisome subunits at 2-min intervals. During replisome assembly, both subunits appeared nearly simultaneously (Fig. 1). In contrast, the DnaN-mCherry focus disappeared up to 10 min after alpha-EYFP disassembly in most (98%) cells (Fig. 1). Thus, our data confirmed that the sliding clamp had a longer association period with the DNA template than the alpha subunit.

**Figure 2:**
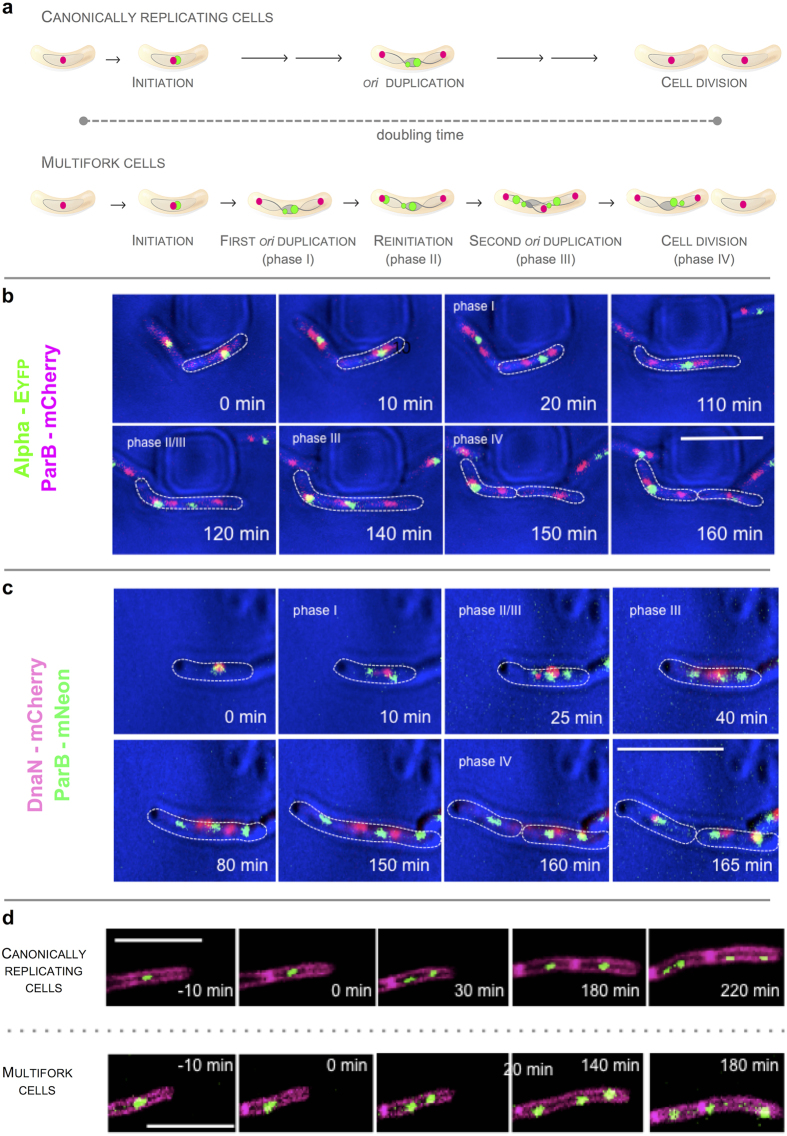
Multifork DNA replication in mycobacteria. (**a**) Replisome patterns of canonically replicating and multifork cells. In most (90%) non-canonically replicating cells, only one of the two previously segregated origins (*oriC*) undergoes a second duplication. Chromosomes within cells (gray line), *oriC*/ParB (magenta dot) and replisomes (green dot) are shown schematically. Examples of cells from different cell cycle phases (**I-IV**) are shown below (**b** and **c**). (**b** and **c**) Replisome dynamics in cells with overlapping rounds of DNA replication. TLFM of cells expressing alpha-EYFP (green) (**b**) or DnaN-mCherry (magenta) (**c**). The *oriC* region was visualized using ParB protein fused to mCherry (**b**) or mNeon (**c**). Scale bar, 5 μm. **d**, Origin localization in canonically replicating and multifork cells. TLFM of cells expressing ParB-mNeon (green focus). Cell walls were stained with FM 5–95 (styryl dye, magenta color). Scale bar, 5 μm.

Because both the DnaN-mCherry and alpha-EYFP strains have some limitations (longer association with the DNA and delayed DNA synthesis, respectively), parallel studies were performed using fluorescent replisome markers. We tracked the spatiotemporal dynamics of replisomes at different time intervals. The dynamics of alpha-labeled replisomes (Supplementary Movie 1) were similar to those previously described for DnaN-FP-marked replisomes^17^. Our TLFM analysis of both reporter strains revealed that a relatively large fraction of cells (more than 10% of each) contained multiple alpha- or DnaN-FP foci and that these were often widely separated (Supplementary Fig. 1c). A careful examination of cells with widely separated foci revealed that the appearance of new alpha- or DnaN-FP-marked replisomes preceded the disappearance of earlier foci, suggesting the onset of another round of replication (see Fig. 2a–c and Supplementary Movies 2 and 3). For DnaN-FP, we only considered cells in which an additional replication round began a minimum of 10 min before the previous replisome spot(s) disappeared (i.e., longer than the interval between the disappearances of alpha-EYFP and DnaN-mCherry).

The initiation of a new round of replication should be followed by the subsequent separation of daughter *oriCs*, which can be monitored by visualizing ParB complexes. As ParB-DNA complexes do not disassemble at any stage of the cell cycle, ParB-FP represents a reliable marker of *oriC*^16^,^17^,^22^. To directly monitor the duplication of *oriC* regions, we replaced the *parB* gene with a *parB-mneon* or *parB-mcherry* fusion gene in the DnaN-mCherry or alpha-EYFP strains, respectively. In canonically dividing cells, the ParB-FP focus will duplicate once per cell cycle, soon after the appearance of DnaN-mCherry (or alpha-EYFP). Thereafter, both nascent foci move towards the cell poles (Fig. 2a). In multifork cells (Supplementary Movies 4 and 5) during the first round of replication, we observed that the *oriC* is duplicated (similar to cells with non-overlapping rounds of replication) almost immediately after replisome appearance (phase I in Fig. 2a–c); the sister *oriC*s segregate to positions near the cell poles. In these cells, however, we observed that an additional replisome focus appeared at one of the segregated *oriC*s (phase II); there was a subsequent additional *oriC* duplication event (phase III). In a substantial fraction of these reinitiating cells (90%), only one of the two previously segregated *oriC*s underwent a second duplication event, which occurred several frames (taken at 5- or 10-min intervals) before the first round of replication terminated (i.e., the first replisome disappeared). As a consequence of this asynchronous reinitiation, three *oriC* regions (i.e., ParB foci) were observed per single cell: two near the cell poles and a third close to the midcell (see Fig. 2a–c). In the remaining 10% of reinitiating cells, both sister *oriC*s were reduplicated. We observed a larger fraction of multifork cells in the alpha-EYFP/ParB-mCherry strain (18%, n = 205) than in the DnaN-mCherry/ParB-mNeon strain (11%, n = 417). Presumably, this difference indicates that the C-period is longer and the B and/or D periods are shorter in the alpha-EYFP strain compared to the DnaN-mCherry strain. Together, our findings clearly demonstrate that in a subset of *M. smegmatis* cells, a new round of replication begins before the previous one has terminated. Interestingly, in mycobacteria only one of the two sister *oriC* regions is typically reduplicated, while in fast-growing bacteria such as *E. coli* and *B. subtilis*, reinitiation is triggered at both *oriC*s.

To exclude the possibility that the fusion of a fluorescent protein with a replisome subunit could affect replication dynamics and trigger the observed reinitiation, we examined this phenomenon using a strain that expressed only the ParB-mNeon fusion. Staining with the membrane-specific dye FM5–95 revealed that even though most dividing cells (89%) had two ParB-mNeon foci, there were a number of cells (11%) in which additional duplication(s) of ParB foci preceded septum formation, usually resulting in three distinct ParB-mNeon foci per cell (Fig. 2d). The division of mother cells with three ParB foci usually gave rise to two daughter cells of unequal size: one with a single ParB complex and one with two ParB complexes (Fig. 2a–d). Thus, one cell inherited a complete chromosome, while the other contained two partially replicated chromosomes (phase IV in Fig. 2a–c).

**Figure 3:**
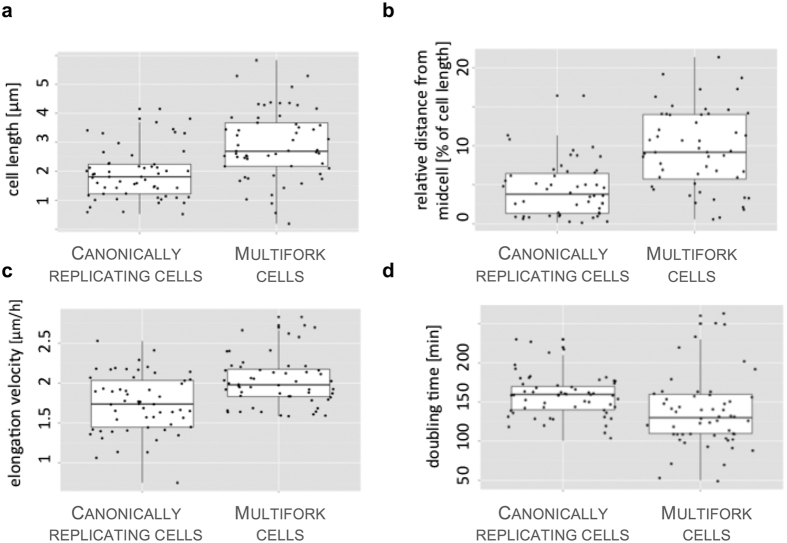
Comparison of various features between canonically replicating and over-replicating cells. (**a**) Cell length. (**b**) Shifting of the division site with respect to the midcell. (**c**) Elongation velocity. (**d**) Doubling time (n = 50). Doubling time was measured as time between two subsequent “snapping” events.

The replication reinitiation observed in a fraction of *M. smegmatis* cells raises an interesting question: Do these cells exhibit different growth and/or division patterns? A closer look revealed that cells exhibiting multifork replication were longer at birth than their non-reinitiating counterparts (5.9 μm vs. 4.9 μm, respectively, p = 1.72e-06 by t-test; Fig. 3a). In addition, septum placement was more often asymmetric (p = 6.84e-08; Fig. 3b), the elongation velocity was greater (2.0 μm h^−1^ vs. 1.7 μm h^−1^, respectively, p = 1.818e-05 by t-test; Fig. 3c), and the doubling time was shorter (136 vs. 158 min, respectively, p = 0.0030; Fig. 3d) in multifork-replicating cells than in cells with non-overlapping C-cycles. When we compared the duration of the C-period in cells that undergo multifork replication with the corresponding period in the mother cell (i.e., from the previous generation), we failed to detect a statistically significant difference between the two groups. Thus, reinitiation was not triggered in daughter cells as a means to compensate for a longer replication time in the mother cell. As it was difficult to observe the dynamics of multiple foci within a single cell, we could not exclude the possibility that reinitiation may compensate for elongated replication in the same generation in which a multifork event occurs. Analyses of the growth parameters of reinitiating cells suggested that this reinitiation might be advantageous: cells with overlapping C-cycles grow faster and divide more frequently than normal cells.

In *E. coli* and *B. subtilis*, multifork replication occurs under optimal temperature and nutrient conditions^3^,^23^,^24^. To test whether these parameters are also crucial for the occurrence of over-replication in *M. smegmatis*, we reduced the temperature in the TLFM analyses. As expected, the time required for chromosome replication was found to be longer at lower temperatures when we compared the C-period obtained at 37 °C (120 +/−9 min) with those obtained at 30 °C (170 +/−16 min) and 25 °C (226 +/−24 min) (Table 1). However, we still observed multifork replication at lower temperatures. Surprisingly, the highest fraction of cells with overlapping C-periods was observed at the lowest tested temperature (25% of cells at 25 °C vs. 11% at 37 °C). At both non-optimal temperatures, cells with overlapping C-cycles were significantly longer and had a significantly higher elongation velocity compared to their normally growing counterparts (p < 0.005 for all comparisons; Supplementary Fig. 1d). We also analyzed the replication dynamics of cells grown in minimal medium (M9 broth supplemented with 0.05% Tyloxapol and 0.1% glycerol) at 37 °C. Even under such conditions, we still observed multifork replication (data not shown), although the fraction of reinitiating cells was lower (6%) than the corresponding fraction of cells growing in rich medium (11%). Thus, unlike *E. coli*, mycobacteria do not appear to employ replication reinitiation exclusively under optimal growth conditions.

Our observation that there is cell-to-cell variation in the replication dynamics of *M. smegmatis* raises the following question: Is this heterogeneity generated by stochastic or deterministic mechanisms? This is particularly interesting considering the peculiar mode of growth and division of mycobacteria, which results in daughter cells that differ in both size and elongation rate. A recent study indicated that there is a deterministic component to this cell-to-cell heterogeneity in size and elongation rate^13^. However, we observed only minor differences in the multifork replication frequencies between cells that inherited new and old poles (57% vs. 43%, respectively). Thus, our data suggest that chromosome replication reinitiation may be a random process in *M. smegmatis*. Moreover, we speculate that asynchronous reinitiation may be a consequence of competition between sister cell origins for limiting replication factor(s), such as proteins responsible for replication initiation and/or pools of precursors that are required for DNA synthesis in slow-growing bacteria. Asynchronous reinitiation may also be a consequence of the absence of a sequestration mechanism. We cannot exclude the possibility that heterogeneity in replication dynamics may be a consequence of relaxed cell cycle checkpoints. It may be tempting to speculate that such relaxation may be associated with a mycobacterial ability to enter a dormant state and/or return to active cell division.

In summary, we unexpectedly discovered multifork replication in a slow-growing bacterium, namely *M. smegmatis*. This phenomenon is even more intriguing when we consider that under optimal growth conditions, the doubling time is longer than the C-period. Moreover, our studies demonstrate for the first time that a clonal population of *M. smegmatis* exhibits cell-to-cell heterogeneity in replication dynamics: some cells exhibit multifork replication, while others do not. Surprisingly, we observed a larger fraction of over-replicating cells under unfavorable growth conditions compared to optimal conditions (24% vs. 11% when grown at 25 °C and 37 °C, respectively). Over-replicating cells extended more quickly and had shorter doubling times than canonically dividing cells. We speculate that the cell-to-cell heterogeneity in chromosome replication dynamics may result from relaxed cell cycle checkpoints and that this helps *M. smegmatis* adapt to different environmental conditions (e.g., nutrient limitations or lower temperatures). Our results can be extrapolated to other species of *Mycobacterium*, including *M. tuberculosis*, because most of the cell cycle proteins, including those involved in chromosome replication, are nearly identical. For example, DnaN protein from *M. smegmatis* shares high homology with the corresponding protein from *M. tuberculosis* (99% identity).

## Methods

### Bacterial strains and plasmids

All plasmids used for mycobacterial transformation were propagated in the *E. coli* DH5α strain. Cells were incubated in LB broth or on LB agar plates (Difco) supplemented with proper antibiotic(s) (ampicillin, kanamycin) and/or other compounds (X-Gal, isopropyl-β-D-1-thiogalactopyranoside [IPTG]) according to standard procedures^25^. Mycobacterial strains were cultured either in 7H9 broth or on 7H10 agar (Difco) supplemented with OADC (BD) and 0.05% Tween80 and/or proper antibiotics. Strains, plasmids and primers are listed in Supplementary Table 1.

The allelic replacement of genes encoding the alpha (*Msmeg_3178*) or beta (*dnaN, Msmeg_0001*) subunits of polymerase DNA III with either *alpha-eyfp* or *dnaN-mcherry* fusion genes, as well as the replacement of the *parB* gene with a *parB-mneon green/parB-mcherry* fusion gene, was performed as previously described^26^. The *eyfp* gene with a short DNA fragment that encoded a 10-amino acid sequence at the 5ʹ-terminus was PCR-amplified using the primers L_EYFP_Eco_Fw and L_EYFP_STOP_Nhe_Rv. Flanking sequences containing the *Msmeg_3178* gene were amplified using mc^2^ 155 (WT) chromosomal DNA as a template and the primer pairs alpha1_Bam_Fw and alpha1_Bsr_Nhe_Rv and alpha2_Bsr_Nhe_Fw and alpha2_Pac_Rv (Supplementary Table 1). The latter two products were cloned into a p2NIL (kan^*R*^) vector, and an EYFP-encoding fragment was inserted at the EcoRV and NheI restriction sites. The obtained plasmids were verified by sequencing. Finally, the pGoal17 cassette was cloned into the *Pac*I site of the p2NIL derivative. The p2NIL*dnaN-mCherry-pGoal* plasmid was constructed as previously described^17^. To prepare the p2Nil*parB-mneon* vector, the *mneon green* gene preceded by a 19-aa encoding linker was amplified from pVV04 (kindly provided by Dr. R. Reyes-Lamothe) using the primers KK_Neon_Fw and KK_Neon_Rv. Next, the vector p2NIL*parB-mcherry*^27^ was amplified with the primers KK_ParBNeon_Fw and KK_ParBNeon_Rv. Gibson Assembly (NEB) was used to assemble the two PCR products, and the pGoal17 cassette was cloned into the PacI site of the p2NIL derivative. *M. smegmatis* cells were transformed with 100 ng of NaOH/EDTA-treated plasmid DNA, and the unmarked mutants were selected as previously described^28^.

The alpha-EYFP and ParB-mNeon strains were generated by transformation with p2NIL *alpha-EYFP-pGoal* and p2NIL-*parB-mNeon-pGoal*, respectively. The alpha-EYFP/DnaN-mCherry and ParB-mNeon/DnaN-mCherry strains were obtained by transforming the alpha-EYFP and ParB-mNeon strains with p2NIL *DnaN-mCherry-pGoal*. Alpha-EYFP/ParB-mCherry was obtained by transforming alpha-EYFP with *p2NILparB-mCherry-pGoal*^27^. The correct allelic replacement and incorporation of integration vectors were confirmed via PCR, Southern blotting and Western blotting. The fusion of the functional fluorescent protein was confirmed using semi-native SDS-PAGE followed by analysis on a Bio Rad PharosFX-Plus Molecular Imager. Western blotting was performed with polyclonal anti-mCherry and/or monoclonal anti-GFP antibodies (Santa Cruz Biotechnology) using standard procedures^29^.

### Microscopy

For live-cell snap-shot imaging, *M. smegmatis* was grown to mid-log phase (OD_600_ = 0.5) in liquid medium, centrifuged (8000 rpm, 5 min), resuspended in PBS, and smeared onto microscope slides. The samples were dried, mounted on coverslips using 5 μl of PBS-glycerol (1:1) solution, and visualized using a Delta Vision Elite imager (GE Healthcare) equipped with softWoRx software (provided with GE Healthcare equipment). Analyses were performed using the “R”^30^ and FIJI software platforms (Image J; https://imagej.nih.gov). For all measurements, two-sided, parametric Student’s t-test was applied. To avoid generation of false assumptions in the case of non-normal distributions, the statistical significance of differences in measured values was confirmed with the non-parametric two-sided Wilcoxon test with minimum 0.995 confidence intervals. For time-lapse observations of the ParB-mNeon strain stained with FM5–95 (membrane dye), cells in mid-log phase were spread on 7H10 agar (supplemented with 10% OADC, 0.5% glycerol, and 0.25 μg/ml FM5–95) and placed in an ibidi 35-mm dish (ibidi GmbH, Germany). Images were acquired at 10-min intervals at 37 °C.

### Microfluidic experiments

TLFM was performed as previously described^17^ using the CellASIC Onix platform (Merck Millipore). For all experiments, 1.5 psi pressure was applied to the incubation plate. The data were collected using a Delta Vision Elite imager equipped with SoftWorx software and analyzed with the “R”^30^ and FIJI software platforms.

## Additional Information

**How to cite this article:** Trojanowski, D. *et al*. Multifork chromosome replication in slow-growing bacteria. *Sci. Rep.* **7**, 43836; doi: 10.1038/srep43836 (2017).

**Publisher's note:** Springer Nature remains neutral with regard to jurisdictional claims in published maps and institutional affiliations.

## Supplementary Material

Supplementary movie 1

Supplementary movie 2

Supplementary movie 3

Supplementary movie 4

Supplementary movie 5

Supplementary Information File

## Figures and Tables

**Table 1:** Cell cycle parameters of alpha-EYFP* and DnaN-FP** strains grown in rich medium at various temperatures.

*M. smegmatis* strain	Temp. [°C]	C period [min]	B + D period [min]	Generation time [min]
Alpha-EYFP*	**37**	145 +/−10	7 +/−15	160 +/−25
DnaN-FP/ParB-mNeon**		120 +/−9	30 +/−12	157 +/−30
	**30**	170 +/−16	40 +/−19	215 +/−26
	**25**	226 +/−24	52 +/−22	274 +/−29

*The alpha-EYFP and alpha-EYFP/ParB-mCherry strains were equivalent for the listed parameters; n = 100. **The DnaN-FP (mCherry or EGFP) and ParB-mNeon strains were equivalent for the listed parameters; n_**37**_ = 84, n_**30**_ = 62, n_**25**_ = 61. Generation time was measured as the time between two subsequent initiation events (in mother cell and daughter cells). This corresponds to the total of C + B + D duration.
