# Supplementary material for: Multifork chromosome replication in slow-growing bacteria
Source: Sci Rep. 2017 Mar 6;7:43836. doi: 10.1038/srep43836 (PMC5338351; doi:10.1038/srep43836)
Supplement: Supplementary Information File [file srep43836-s6.pdf]

Supplementary info file

Multifork chromosome replication in slow-growing bacteria

Damian Trojanowski[1], Joanna Hołówka[2], Katarzyna Ginda[1,3], Dagmara Jakimowicz[1,2], and Jolanta Zakrzewska-Czerwińska[1,2*]

Supplementary Table 1

Plasmids, strains and oligos used in this study

**Strains**		
**Strain**	**Relevant genotype**	**Source**
WT	*M. smegmatis* Mc^2 155	Laboratory stock
DnaN-EYFP (JH01)	*M. smegmatis* Mc^2 155 *dnaN-egfp*	[17]
DnaN-mChery (DT05)	M. *smegmatis* Mc^2 155 *dnaN-mcherry*	[17]
ParB-mNeon	*M. smegmatis* Mc^2 155 *ParB-mNeon*	This study
Alpha-EYFP	*M. smegmatis* Mc^2 155 *alpha-eyfp*	This study
Alpha-EYFP/DnaN-mCherry DnaN-mCherry	*M. smegmatis* Mc^2 155 *alpha-eyfp, dnaN-mcherry*	This study
ParB-mNeon/DnaN-mCherry	*M. smegmatis* Mc^2 155 *ParB-mNeon, dnaN-mcherry*	This study
Alpha-EYFP/ParB-mNeon	*M. smegmatis* Mc^2 155 *alpha-eyfp, ParB-mNeon*	This study
**Primers**		
**Name**	**Sequence 5' to 3'**	**Tm [˚C]**
L_EYFP_Eco_Fw	CCGATATCCTGCCGGGCCCGGAGCTGCCGGGCCCGGAGATGGTGAGCAAGGGCGAG	58
L_EYFP_STOP_Nhe_Rv	CCGCTAGCTTACTTGTACAGCTCGTCC	55
alpha1_Bam_Fw	CCGGATCCAGTTCACCGACTTCTCGGAC	62
alpha1_Bsr_Nhe_Rv	CCTGTACAGATATCGCCGAGGCAGCCGGGG	60
KK_Neon_Fw	TCGGCTGGCTCCGCTGC	62
KK_Neon_Rv	TTATTTGTACAATTCATCCATGCCC	61
KK_ParBNeon_Fw	GGGCATGGATGAATTGTACAAATAAAAGTCGGTGTCAGCGGACG	62
KK_ParBNeon_Rv	GCAGCGGAGCCAGCCGAACTCGTTCTGGGCGCTCATCA	62
alpha2_Bsr_Nhe_Fw	CCTGTACAGCTAGCCTTTTTCGCCGGGGATCTGC	62
**Plasmids**		
**Name**	**Plasmid features**	**Reference**
pGoal17	ampicillin resistance, *oriE*, containing selective PacI cassete with *lacZ*, *sacB* and *kanR* genes	[26]
p2NIL	kanamycin resistance, *oriE*, suicide vector for allelic replacement	[26]
p2NIL-DnaN – mCherry-pGoal	kanamycin resistance, *oriE*, *dnaN-mcherry* fusion gene, PacI cassete	[17]
p2NIL*ParB-mNeon-pGoal*	kanamycin resistance, *oriE*, *parB-mneon* fusion gene, PacI cassete	This study
p2NIL*ParB-mCherry-pGoal*	kanamycin resistance, *oriE*, *parB-mcherry* fusion gene, PacI cassete	[27]
p2NIL*alpha-eyfp-pGoal*	kanamycin resistance, *oriE*, *alpha-eyfp* fusion gene, PacI cassete	This study

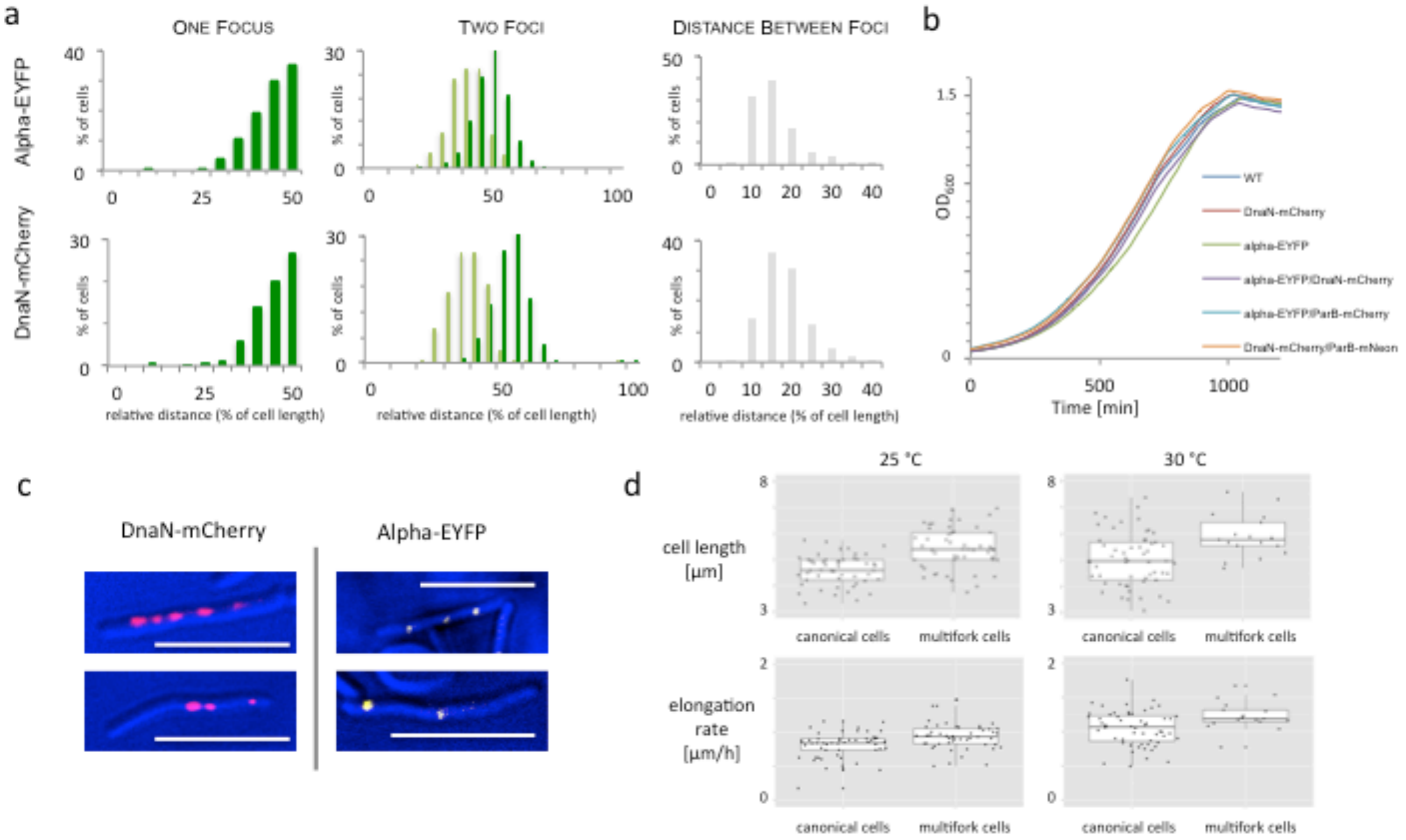

Supplementary Fig. 1. Characteristics of the strains analyzed

a, Positions of one focus and two foci (in relation to the nearest pole) and the distance between two foci ($n_{\mathbf{DnaN\text{-}mCherry}}$=1,700, $n_{\mathbf{alpha\text{-}EYFP}}$=525). **b,** Growth curves of the strains used in this study. **c,** Examples of multifork cells. Scale bar, 5 µm. **d,** Comparison of cell lengths and elongation rates between canonically replicating (n_{25}=50, n_{30}=50) and over-replicating (n_{25}=50, n_{30}=17) cells.

Movies

1. Supplementary Movie 1

A representative TLFM movie of the alpha-EYFP strain. Fluorescent spots represent alpha subunits tagged with enhanced yellow fluorescent protein. Images were acquired automatically every 10 min.

2. Supplementary Movie 2

A representative TLFM movie of the alpha-EYFP strain, showing multifork cells. Arrows indicate the occurrence of additional replisome assembly during an ongoing

round of replication. Additional replisomes assemble around the midcell of future daughter cells. Images were acquired automatically every 10 min.

3. Supplementary Movie 3

A representative TLFM movie of the DnaN-mCherry strain, showing multifork cells. Arrows depict the occurrence of additional replisome assembly during an ongoing round of replication. A cell in which a multifork event first occurred will continue to grow in multifork mode for several generations. Images were acquired automatically every 10 min.

4. Supplementary Movie 4

A representative TLMM movie of the DnaN-mCherry/ParB-mNeon strain, showing multifork cells. Arrows indicate duplications of ParB-mNeon foci followed by additional duplication of one of nascent oriC within a single cell cycle. Images were acquired automatically every 10 min.

5. Supplementary Movie 5

A representative TLMM movie of the alpha-EYFP/ParB-mCherry strain, showing multifork cells. Arrows depict duplications of ParB-mCherry foci followed by additional duplication of one of nascent oriC within a single cell cycle. Images were acquired automatically every 10 min.
